# Classification of four ovine breeds of southern peninsular zone of India: Morphometric study using classical discriminant function analysis

**DOI:** 10.1186/2193-1801-2-29

**Published:** 2013-01-29

**Authors:** Dinesh Kumar Yadav, Anand Jain, Viswanath Sheshagirirao Kulkarni, Mandera Giriyappa Govindaiah, Thimmappa Aswathnarayan, Devinder Kumar Sadana

**Affiliations:** 1 National Bureau of Animal Genetic Resources, G. T. Road, Baldi By-Pass, Karnal, 132 001 Haryana India; 2 University of Agricultural Sciences, Dharwad, 580 005 Karnataka India; 3 KVAFSU, Veterinary College, Bangalore, 560 024 Karnataka India; 4 Department of Animal Husbandry and Veterinary Services, Bangalore, 560 001 Karnataka India

**Keywords:** Bellary, Discriminant analysis, Morphometric traits, Hassan, Kenguri, Mandya, Sheep

## Abstract

Six morphometric traits (height at withers, body length, chest girth, ear length, tail length and body weight) were analyzed to characterize from a breed point of view 1981 sheep from four ovine breeds (Bellary, Kenguri, Hassan and Mandya) of southern peninsular zone of India. Discriminant Function Analysis was used to distinguish between four breeds by morphometric traits. The population variability showed Kenguri ewes were the largest and heaviest followed by Bellary, Hassan and Mandya whereas Kenguri rams were followed by Bellary, Mandya and Hassan. Overall sexual dimorphism (m/f) was 1.13, with Kenguri males being 47% heavier than females. The coefficient of variation of all traits in four breeds ranged from 4.06% to 30.28%. The flocks and age effects showed a high heterogeneity among females of different flocks. Height at withers was most discriminating trait in separating the four sheep breeds. The Mahalanobis distance of the morphological traits between Kenguri and Mandya sheep was most while the least differentiation was observed between Kenguri and Bellary sheep. Nearest neighbour discriminant analysis showed that most Kenguri sheep were classified into their source population followed by Mandya. However, varied percentages of misclassification between different breeds were observed showing the level of genetic exchange that has taken place between the breeds overtime. UPGMA based dendrogram showed formation of two separate groups; Mandya and Hassan clustered together while Bellary and Kenguri formed other group.

## Introduction

India is a rich source of diverse ovine germplasm with 74 million sheep which is 6.8% of world sheep population (FAOSTAT 2010). According to the FAO (2000), there are 60 sheep breeds in India including well-recognized, lesser known and some wild species. Karnataka, an Indian state lying in its Southern peninsular agro-ecological zone, has sheep as a socio-economically important livestock reared primarily as a source of mutton in rural areas. The state enjoys tropical monsoon type climate and hosts four well adapted sheep breeds viz. Bellary, Kenguri, Hassan and Mandya. Mandya breed is perhaps the best mutton breed of the country as far as conformation is concerned, although body weights, weight gains, feed conversion efficiency and carcass yield are not very superior to most other breeds (Acharya 1982).

Contributions to characterization of local domestic animal populations are of major importance as the breed is the operation unit for the assessment of livestock diversity all over the world (Simon 1999; Duchev and Groeneveld 2006). While Lanari *et al*. (2003) emphasized characterization of livestock breeds as the first approach to a sustainable use of animal genetic resources; Gizaw *et al*. (2007) highlighted identification of populations based on morphological descriptors as the first phase of characterization. Despite the fact that, in general, no breed classification should rely exclusively on biometric data, these play a proxy or complementary role in the description of a breed (Parés i Casanova 2009). Attempts for phenotypic characterization and classification of Indian sheep breeds have not only been limited to single breed/population but also to the use of univariate analysis methods (Bohra *et al*. 1993; Sahana *et al*. 2001; Kumar *et al.*^2006^; Singh *et al*. 2007; Jain *et al*. 2009; Yadav *et al*. 2009,2011,2012). The current trend in livestock classification using multivariate statistical tools (Herrera, 1996; Zaitoun *et al*. 2005; Dossa *et al*. 2007; Traoré *et al*. 2008; Yakubu *et al*. 2011) has not yet found place in the morphometric differentiation of Indian sheep breeds. In all these cases, the classical discriminant function analysis method was found to be relatively efficient and allowed differences between breeds and subpopulations to be detected, as well as the relative distance between them to be assessed. Further, the variables with greatest power to differentiate were identified enabling them to be weighted accordingly. Our study, attempts to investigate the above aspects in four Indian sheep breeds based on morphometric traits using multivariate analysis methods.

## Materials and methods

### Study area and agro-climatic conditions

Indian sheep breeds are distributed in the four major agro-ecological regions: the Northern temperate, North-western arid and semi-arid, Southern peninsular and Eastern (Acharya 1982; Bhatia and Arora 2005). The present study was conducted in the Karnataka state located between latitudes 11°30' N and 18°23' N and longitudes 74°05' E and 78°35' E in the Sothern peninsular region of India. Karnataka is the third largest sheep rearing state of India with 9.5 million sheep population (BAHS 2010). The climate is tropical-monsoon type with temperature reaching as high as 42°C in summers and as low as 12°C in winters. The breeding tract of Bellary and Kenguri breeds fall in Northern Dry Zone of the state, a drought prone area. Annual rainfall varies from 633–807 mm and temperature ranges from 18°C to 40°C. The altitude ranges from 465–786 meters above sea level. The soils are red sandy loam, red loam and shallow black. Major crops grown are sorghum (*Sorghum bicolor*), Groundnut (*Arachis hypogea*), Bajra (*Pennisitum typhodes*), oilseeds and pulses. The distribution areas of Hassan and Mandya sheep fall in Southern Transition Zone of Karnataka. The altitude varies from 150–250, 250–300 and 300–500 meters above sea level at different places. Annual rainfall ranges from 671–887 mm. Majority of soils are red sandy loam but in some pockets red loams are also present. Crops grown in the area are sorghum (*Sorghum bicolor*), ragi *(Eleusine coracana)*, maize (*Zea mays*), groundnut (*Arachis hypogea*), sugarcane (*Saccharum officinarum*), cotton (*Cocos nucifera*) and arecanut (*Areca catechu*).

### Management practices

All sheep breeds under study were subjected to extensive management system with little or no supplementary feeding. The animals grazed during the day on crop stubbles and low grade forage on barren land and roadsides for 6–8 hours and walked a distance of about 5–20 kilometres. They were taken to water source two or three times a day. During lean period, only Mandya sheep were given supplementary feed like jowar (*sorghum*), wheat (*Triticum*), ragi (*Eleusine coracana*), horse gram (*Macrotyloma uniflorum*) husk, and leaves of acacia, peepal (*Ficus religiosa*) and neem (*Azadirachta indica*). Stall feeding of small flocks was a routine practice carried by Mandya sheep rearers but Bellay sheep rearers provided supplementary feeding to the breeding rams during breeding season only. Mandya and Kenguri sheep flocks were stationary. Most of the Hassan sheep flocks were stationary but some flocks migrated to the adjoining areas during the lean periods of March to June. Bellary sheep migrate to the southern parts of the state (up to 150–300 kilometres) during December-August. Some of the Kenguri sheep farmers introduced Bellary rams in their flocks, and admixtures of the two sheep breeds were observed. Adequate health care was provided. Animals were vaccinated against haemorrhagic septicaemia, enterotoxaemia and sheep pox. Anthelmintics were used against the endoparasites. Adult mortality in the four breeds ranged from 5–15%. Flock size of the four breeds varied from small to large. Majority of the Bellary sheep flocks were large to medium sized having average flock size 143 (5 adult males, 106 adult females and 32 young). Most of the Kenguri sheep flocks were medium sized comprising average flock size 87 (4 adult males, 60 adult females and 23 young). Hassan sheep flocks were medium sized consisting average flock size 34 (3 adult males, 23 adult females and 8 young). Flock purity in Bellary, Kenguri and Hassan sheep was approximately 95 percent. Mandya sheep flocks were generally small and in pure form. Average flock size was 16 (1 adult male, 11 adult females and 4 young). At night sheep flocks were housed in a shed thatched with tree branches and crop by-products with a fenced enclosure made of bushes or locally available material or iron wires. The sheep paddocks were either a part of owner’s dwelling or neighbouring to it (Jain *et al*. [Bibr CR13_82][Bibr CR14_82][Bibr CR15_82][Bibr CR16_82]).

### Data collection

**Figure 1:**
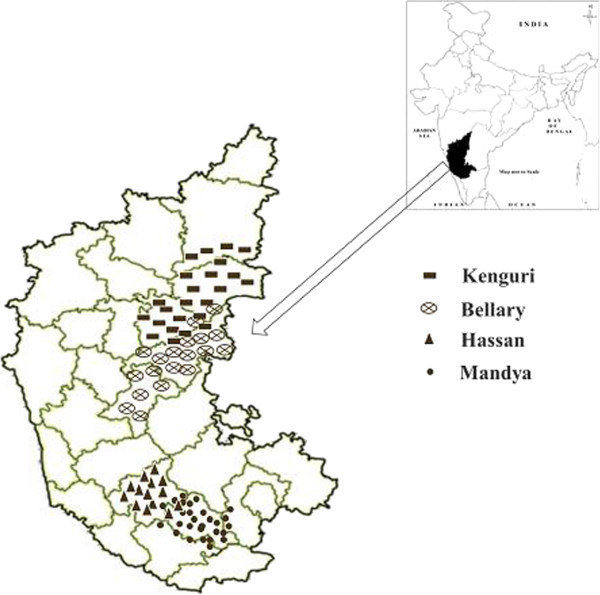
**Breeding tract of the four sheep breeds of Karnataka state.**

Bellary sheep are distributed in Bellary, Davangere districts and the adjoining areas of Haveri and Chitradurga districts. These are of strong built, medium to large sized with coat colour ranging from white through various combinations of white and black to complete black. Kenguri sheep prevails primarily on Koppal and Raichur districts, are large-sized with dark brown (coconut colored) body color. Hassan sheep are small to medium sized having white body colour with light brown or black spots. The breed exists in Hassan district. Mandya sheep are relatively small in size. Coat colour is white with light brown face usually extending up to neck. They have compact and low set body with a typical reversed U-shape conformation from the rear, mainly distributed in Mandya district and bordering area of Mysore and Bangalore rural districts of Karnataka (Figure 1). Total number of sheep during the year 2003 within Bellary, Kenguri, Hassan and Mandya distribution areas were 1.516, 0.571, 0.196 and 0.34 million respectively. The corresponding figures in the year 2007 were 2.187, 1.035, 0.201 and 0.382 million (BAHS 2010). While breed-wise population statistics are not in vogue, above figures indicate an increase in total number of sheep in the distribution area of respective breeds during the period 2003–2007. Sampling was carried out from the farmer’s flocks in the distribution area on a total of 327 flocks (Bellary, 116; Kenguri, 86; Hassan, 62; Mandya, 63) selected at random. Morphological measurements were taken on 1981 female (f) and male (m) animals (Bellary-576 f, 148 m; Hassan-205 f, 54 m; Kenguri-416 f, 82 m and Mandya-446 f, 54 m). All studied animals were from two teeth to eight teeth of age, which was estimated from their dentition. The six morphometric traits measured were height at withers (HW), body length (BL), chest girth (CG), ear length (EL), tail length (TL) and body weight (BW). The body weights (kilogram) were recorded with a weighing machine. The other traits were measured with a measuring tape (centimeter) after making the animal stand squarely on an even ground.

### Statistical analysis

Descriptive statistics for the morphometric traits were obtained using JMP Genomics 4.1 software of SAS (2009). Means separation was done using Tukey’s HSD of the same statistical package at 5% significance level. The influence of age and flock on the body traits measured was assessed fitting a model which includes the age effect with 4 levels (2-teeth, 4-teeth, 6 teeth and 8-teeth) and flock effect with different levels in four breeds (Bellary, 116; Kenguri, 86; Hassan, 62; Mandya, 63). The sexes were analyzed separately due to differences in sample size and, to the high sexual dimorphism found. Stepwise discriminant procedure was applied using PROC STEPDISC to determine which morphological traits have more discriminant power than others. The relative importance of the morphometric variables in discriminating the four breeds of sheep was assessed using the level of significance (p < 0.05) and partial R^2^ values ≥0.01. The CANDISC procedure was used for calculating the Mahalanobis distances of the morphological traits, and derived canonical functions. The DISCRIM procedure was used to assign each individual sheep to its breed. Mahalanobis distances generated during the canonical discriminant analysis were used to construct a dendrogram using the unweighted pairs group method analysis (UPGMA).

## Results

**Figure 2:**
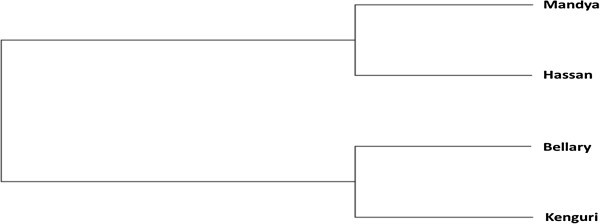
**UPGMA tree based on pair-wise Mahalanobis distances of four sheep breeds.**

Means alongwith coefficient of variation for the analyzed morphometric traits of both sexes of four breeds are provided in Table 1. One-way ANOVA determined statistically significant differences between breeds. Amongst the four sheep breeds, Kenguri ewes were the largest and heaviest followed by Bellary, Hassan and Mandya ewes whereas in rams the order was Kenguri followed by Bellary, Mandya and Hassan. The measure of sexual dimorphism (m/f) has also been included in Table 1 to express these differences between males and females. Kenguri males were 47% heavier than females, as were values of other traits in all breeds, except for EL which demonstrated no significant differences. The coefficient of variation of all traits in four breeds ranged from 4.06-30.28 percent (females) and 4.77- 27.29 percent (males). Breed-wise average varied roughly from 9–12.5 percent whereas the overall coefficient of variation of all breeds was 11 percent (males) and 10.4 percent (females). Table 1 also shows the significance of the age of the animal and flock effects on the analyzed traits. Sexual dimorphism was observed in the morphological traits with males having significantly higher (p < 0.05) BL, HW, CG, TL and BW than females. In females, the flock effect was highly significant (p < 0.001) except EL in Hassan breed while in males it was non-significant except for Bellary breed showing a high homogeneity between flocks. Age effect in females was non-significant for 4, 3, 3 and 1 traits in Bellay, Kenguri, Hassan and Mandya breeds respectively while in males it was non-significant except 4 traits of Bellary breed. The results of the stepwise discriminant analysis are presented in Table 2. All the measured variables were found to be significant (p < 0.0001) in both sexes. HW had more discriminant power than the others as indicated by their higher R^2^ and F-values. Therefore, all other variables were removed from the final model (Dossa *et al*. 2007). In the canonical discriminant analysis, the canonical variable (CAN1) generated was significant (*p* < 0.0001) (Table 3). The Mahalanobis distances between the four sheep breeds are presented in Table 4 which may be termed as short, medium and long. Mahalanobis distance between Kenguri and Mandya can be grouped as long while the distance between Bellary and Kenguri, Bellary and Hassan, and Hassan and Mandya breeds as short. The distance between the Bellary and Mandya and Kenguri and Hassan were intermediate. All pair wise distances were highly significant (*p* < 0.0001), indicating that differences between breeds are important. The largest distance was between Kenguri in the northern Karnataka and Mandya in the far south of the state whereas the shortest distance was between Kenguri and Bellay having flanking breeding tracts. The dendrogram (Figure 2) shows formation of two separate groups, Mandya and Hassan clustering together while Bellary and Kenguri forming another group. Percent classification of individual sheep into four genetic groups is shown in Table 5. In females, most Kenguri sheep (75.72%) were classified into their source population followed by Mandya (72.65%). However, while 26.23% of Mandya ewes were misclassified as Hassan ewes, 16.1% of Hassan ewes were wrongly assigned as Mandya ewes. Similarly in rams, 38.89% of Hassan were misclassified as Mandya, 33.33% of Mandya were wrongly assigned as Hassan rams. Nevertheless, an average of 70 percent females and 67 percent males for the four breeds were correctly assigned into their genetic groups.

**Table 1:** **Means, coefficient of variation, and significance of flock and age effects for each of the morphological traits in different sheep breeds**

	Body Traits	Females				Males				Sexual dimorphism (m/f)
		Statistics		Fixed effects		Statistics		Fixed effects		
		Mean	CV	Flock	Age	Mean	CV	Flock	Age	
Bellary	BL	65.6^b^	5.27	***	NS	70.2^f^	6.73	***	***	1.07
	HW	68.9^b^	4.61	***	NS	74.5^f^	5.37	***	***	1.08
	CG	76.0^b^	5.43	***	***	82.5^f^	6.25	***	***	1.08
	EL	13.2^c^	30.28	***	NS	13.3^f^	27.29	*	NS	1.00
	TL	11.2^a^	13.04	***	NS	12.5^e^	12.64	***	NS	1.12
	BW	31.8^b^	16.55	***	***	43.0^f^	17.54	***	***	1.35
Kenguri	BL	67.3^a^	4.48	***	***	74.7^e^	5.40	NS	NS	1.11
	HW	72.6^a^	4.06	***	NS	81.5^e^	4.77	NS	NS	1.12
	CG	78.4^a^	5.22	***	***	89.4^e^	5.50	NS	NS	1.14
	EL	15.2^a^	9.45	***	NS	15.2^e^	8.51	NS	NS	0.99
	TL	10.1^bc^	16.26	***	NS	12.0^ef^	16.32	NS	NS	1.19
	BW	35.9^a^	13.72	***	***	52.6^e^	14.87	NS	*	1.47
Hassan	BL	62.0^c^	5.07	**	**	64.3^h^	7.49	NS	NS	1.04
	HW	60.8^c^	5.64	***	NS	64.4^g^	7.15	NS	NS	1.06
	CG	71.9^c^	7.32	***	***	75.2^h^	7.09	NS	NS	1.05
	EL	14.1^b^	10.45	NS	NS	14.1^ef^	9.30	NS	NS	0.99
	TL	10.3^b^	13.26	**	NS	11.5^fg^	20.03	NS	NS	1.11
	BW	28.3^c^	17.09	***	***	32.8^h^	19.22	NS	NS	1.16
Mandya	BL	60.5^d^	6.09	***	**	68.3^g^	5.52	NS	NS	1.13
	HW	54.3^d^	8.15	***	**	62.3^h^	7.12	NS	NS	1.15
	CG	69.3^d^	7.09	***	***	79.1^g^	6.86	**	NS	1.14
	EL	13.0^c^	14.12	***	NS	12.8^f^	13.73	*	NS	0.99
	TL	9.9^c^	11.50	***	*	10.7^g^	13.58	NS	NS	1.08
	BW	26.8^d^	16.32	***	***	37.2^g^	16.78	NS	NS	1.39

BL, body length; HW, height at withers; CG, chest girth; EL, ear length TL, tail length; BW, body weight; CV, coefficient of variation (%); ^abcd^means (female) and ^efgh^means (male) in the same column with different superscripts are significantly different ; *Significance level: p < 0.05; ** Significance level: p < 0.01; *** Significance level: p < 0.0001; Body weights are in kilogram, other traits are in centimetre.

**Table 2:** **Stepwise selection summary of traits**

Sex	Step	Trait entered	Partial R^2^	F-value	p > F	Wilk’s lambda	P < lambda	Average squared canonical correlation	p > ASCC
Female	1	HW	0.808	2301.53	<0.0001	0.19183	<0.0001	0.269	<0.0001
	2	TL	0.116	72.19	<0.0001	0.16943	<0.0001	0.306	<0.0001
	3	BW	0.094	56.80	<0.0001	0.15346	<0.0001	0.328	<0.0001
	4	EL	0.077	45.63	<0.0001	0.14161	<0.0001	0.348	<0.0001
	5	CG	0.031	17.19	<0.0001	0.13728	<0.0001	0.356	<0.0001
	6	BL	0.015	8.66	<0.0001	0.13513	<0.0001	0.359	<0.0001
Male	1	HW	0.740	317.01	<0.0001	0.25991	<0.0001	0.246	<0.0001
	2	BL	0.223	31.87	<0.0001	0.20193	<0.0001	0.305	<0.0001
	3	TL	0.086	10.47	<0.0001	0.18448	<0.0001	0.331	<0.0001
	4	CG	0.077	9.29	<0.0001	0.17015	<0.0001	0.351	<0.0001
	5	EL	0.066	7.77	<0.0001	0.15892	<0.0001	0.372	<0.0001

**Table 3:** **Standardized coefficients or the canonical discriminant function, the canonical correlation and the eigenvalue**

Traits	Discriminant variate (CAN1)	
	Female	Male
HW	2.28	1.95
Adjusted Canonical correlation	0.899	0.860
Approximate standard error	0.004	0.014
Eigenvalue	4.212	2.847

**Table 4:** Mahalanobis distance between the four sheep breeds*

Breed	Bellary	Kenguri	Hassan	Mandya
**Bellary**	**0**	1.08	5.20	16.84
**Kenguri**	2.85	**0**	11.01	26.43
**Hassan**	5.88	16.92	**0**	3.32
**Mandya**	8.66	21.44	0.27	**0**

*Above diagonal values are of females and below diagonal are of males.

**Table 5:** **Percent (%) of individual sheep classified into four genetic groups**

Sex	Breed	Bellary	Kenguri	Hassan	Mandya
**Female**	**Bellary**	61.81	29.34	8.85	0
	**Kenguri**	24.04	75.72	0.24	0
	**Hassan**	13.66	0.49	69.75	16.10
	**Mandya**	1.12	0	26.23	72.65
	**Error level**	0.38	0.24	0.30	0.27
	**Priors**	0.25	0.25	0.25	0.25
**Male**	**Bellary**	67.57	20.27	12.16	0
	**Kenguri**	13.41	86.59	0	0
	**Hassan**	5.56	1.85	53.70	38.89
	**Mandya**	5.56	0	33.33	61.11
	**Error level**	0.32	0.13	0.46	0.39
	**Priors**	0.25	0.25	0.25	0.25

## Discussion

Sexual dimorphism was observed in the morphological traits with males having significantly higher (p < 0.01) BL, HW, CG, TL and BW than females. The differences between EL were non-significant. These differences might be due to differences in growth rates, metabolic rates, and growth and reproductive strategies. Considerable variation between breeds was found for morphological traits. Amongst the four sheep breeds, Kenguri ewes were the largest and heaviest followed by Bellary, Hassan and Mandya whereas Kenguri rams were the largest and heaviest followed by Bellary, Mandya and Hassan. It is observed that in females Hassan breed is larger and heavier than Mandya whereas in males the situation is reverse. This could be due to adoption of better management practices in Mandya owing to preferential demand of elite rams for breeding due to its better quality mutton. Mandya sheep possess a compact body with a typical reversed U-shape wedge from the rear and are known for its meaty conformation and excellent meat quality (Acharya 1982; Thangaraju 2009; Jain *et al*. 2005b). The mean values (Table 1) would allow for the defining of the adult morphological standard of the four breeds from both the mean point of view as well as variability. The coefficient of variation of all traits in four breeds ranged from 4.06-30.28 percent with only EL, TL and BW higher than 10 percent which corresponds with a mean homogeneity within the breed (Herrera 2007). The overall coefficient of variation of all breeds was slightly higher in males (11.0%) than females (10.4%) indicating the overall lower heterogeneity of females than among males when viewed globally. The flock effect was highly significant (p < 0.001) in females of all breeds while it was non-significant in males of all breeds except Bellary. Sample size could be the possible reason for this exception. The number of males sampled in Bellary was large (148) as compared to other breeds (Kenguri: 82; Hassan: 54; Mandya: 54). In view of significant flock effect females appear different between flocks; the overall heterogeneity of females is even lower than among males when viewed globally. The age had highly significant effect (p < 0.001) only for two (CG and BW) traits, showing a high heterogeneity among females of different flocks while in males it resulted non-significant effects except for Bellary breed showing a high homogeneity between flocks, an indication that possible influencing factors could be a smaller sampling of males and differential treatment that males receive. Legaz *et al.*^2011^ demonstrated similar findings in Assaf sheep and reasoned that fewer males could be an influencing factor in the signification.

The best discriminant function model used in this study included only one morphological measure (HW) out of the six pre-selected. This demonstrates that taking measurements on this trait could be sufficient in differentiating between these four sheep breeds than acquiring numerous other measurements on morphometric traits. Some of the discriminating variables obtained in the present study are similar to earlier findings (Herrera *et al*. 1996; Dossa *et al*. 2007; Yakubu *et al*. 2011) in the morphostructural differentiation of sheep and goat breeds/populations.

The Mahalanobis distances differed between sexes and breeds. The highest distance between Kenguri and Mandya possibly reflect differences in body size. Generally, such phenotypic divergence between breeds/populations might be partly associated with the differences in management practices, agro-climatic conditions and biophysical resources. Relative breeding objectives depending upon the utility of the breed may also act as important factor. Mandya sheep are relatively smaller in size having compact body as compared to Kenguri and Bellary sheep. Therefore, differences in Mahalanobis distances between different populations and sexes are highly likely to exist. Larger the differences in body measures, larger are the distances between the breeds/populations. Similar results were reported in sheep, goats and horses (Dossa *et al*. 2007; Yakubu *et al*. 2011; Yakubu and Ibrahim Isa 2011; Kefena *et al*. 2012). Diversity in morphometric traits between breeds could be due to genetic differences as well as environmental factors and may reflect the level of adaptation of these breeds to specific agro-climatic conditions of their distribution areas. The genetic distance theoretically describes the differences between breeds. Phenotypic comparison based on morphological characters can provide to some extent a reasonable representation of genetic differences among populations (Dossa *et al*. 2007). Phenotypic differences are maintained in part by the reduction of gene flow among populations separated by large distances (Yakubu and Ibrahim Isa 2011). The results indicate that adaptive divergence in morphological traits was higher between Kenguri and Mandya, Bellary and Mandya, and Kenguri and Hassan sheep and lower between Kenguri and Bellary, Hassan and Mandya, and Hassan and Bellary sheep. The higher divergence among breeds that commensurate geographical distance of their respective habitat may be due to reproductive isolation by distance. However, the correlation of morphological divergence with quantitative differences in economically important traits like growth rate, body weight at different ages and carcass characteristics of these breeds may help to formulate strategy for planned breeding in future. The lower divergence may be due to the genetic exchange that has taken place overtime between the breeds owing to their management practices, geographical proximity and intermixing. Kumar *et al*. 2006; Singh *et al*. 2007; Yadav *et al*. 2009,2011 have reported genetic/breed dilution in Indian sheep due to intermixing. Similar results on adaptive divergence in morphological characters during the morphometric differentiation of sheep were reported on Ethiopian sheep (Gizaw *et al*. 2007) and Nigerian sheep (Yakubu and Ibrahim Isa 2011).

The discriminant analysis revealed that an average of 70 percent females and 67 percent males correct classification of the sampled sheep of the four breeds can be achieved using HW variable. This indicates the heterogeneity of the sheep breeds. The misclassification percentages were 24-38% (females) and 13-46% (males). The reason that could be adduced for these misclassifications is intermingling between the four sheep breeds. As evident from Figure 1 and Figure 2, the four breeds group into two clusters viz. northern part of the state (Kenguri and Bellary) and southern part (Hassan and Mandya). The genetic admixture could have been brought about mainly by the activities of pastoralists who moved from one place to another with their flocks in search of pasture and water. Such transhumance has been cited as a dynamic factor of gene exchange (Draganescu 1997), which is also supported by the management practices of these breeds (Jain *et al*. [Bibr CR13_82][Bibr CR14_82][Bibr CR15_82][Bibr CR16_82]). Several other studies have also reported genetic/breed dilution in Indian sheep due to intermixing (Kumar *et al*. 2006; Singh *et al*. 2007; Yadav *et al*. 2009,2011). Close/overlapping body measures of animals of different breeds/groups could also be a factor to the misclassification of animals. Nevertheless, the computed high degree of correct classification of sheep in their respective genetic source group (breed) confirmed the high discriminating power of the morphological measurement (HW). The use of multivariate discriminant analyses therefore could be successfully used in morphometric differentiation of the four sheep breeds. The findings are in consonance with earlier reports on the use of discriminant analysis to classify animals of different genetic groups or ecotypes (Zaitoun *et al*. 2005; Dossa *et al*. 2007; Traoré *et al*. 2008; Yakubu *et al*. 2011). Nevertheless, the method may have a limitation. It is possible that if the phenotypic distributions of a trait are overlapping between two populations, it will not be able to classify such individuals correctly in their source group. Two individuals may be very similar with respect to body weight, body length or tail length, but even then they could be genetically quite distant. Molecular marker information is needed to make the distinction clear. Hence, further characterization using comparative genetic analyses will consolidate information arising from morphological differentiation. Such phenotypic and genetic data when logically combined into a single database could be critical for decisions regarding indigenous sheep breeds’ exploitation and conservation (Kunene *et al*. 2009).

## Conclusions

The study establishes the structure and the degree of variability between the four breeds. The application of univariate and multivariate statistical methods enabled us to discriminate between the sheep breed using morphometric traits. HW was found as the most discriminating trait in separating the breeds. Flock and age effects established high heterogeneity among females of different flocks whereas in males these effects showed homogeneity between flocks except for Bellary breed. High Mahalanobis distance coupled with zero percent misclassification between Kenguri and Mandya indicated negligible genetic exchange between these breeds. The low Mahalanobis distance between Kenguri and Bellary, Hassan and Mandya, and Hassan and Bellary at the morphological level could be attributed to the genetic exchange that has taken place between these sheep breeds. However, in overlapping morphometric traits the method has a limitation which can be corroborated by genetic characterization using genetic markers. Nevertheless, the classical discriminant function analysis has successfully classified the four breeds in distinct groups.
